# Epidemiology, Treatment Patterns, and Outcomes of Metastatic Soft Tissue Sarcoma in a Community-Based Oncology Network

**DOI:** 10.1155/2014/145764

**Published:** 2014-01-12

**Authors:** Clara Chen, Rohit Borker, James Ewing, Wan-Yu Tseng, Michelle D. Hackshaw, Shanmugapriya Saravanan, Rahul Dhanda, Eric Nadler

**Affiliations:** ^1^Department of Information Technology, Health Economics and Outcomes Research, McKesson Specialty Health, The Woodlands, TX, USA; ^2^GlaxoSmithKline, Philadelphia, PA 19112, USA; ^3^Texas Oncology, Dallas, TX, USA; ^4^Baylor Charles A. Sammons Cancer Center, Dallas, TX, USA; ^5^Baylor University Medical Center at Dallas, Dallas, TX, USA; ^6^Texas Oncology, Baylor Sammons Cancer Center, 3410 Worth Street, Dallas, TX 75246, USA

## Abstract

*Purpose*. To assess epidemiology, treatment patterns, and outcomes of metastatic soft tissue sarcoma (mSTS) patients in USA community oncology practices. *Methods*. This retrospective, descriptive study used US Oncology's iKnowMed electronic health records database. Adults (≥18 years) with mSTS and at least two visits between July 2007 and June 2010 were included. Key outcomes were practice patterns, overall survival (OS), and progression-free survival (PFS). *Results*. 363 mSTS patients (174 treated and 189 untreated) met the prespecified exclusion/inclusion criteria. The most common subtypes were leiomyosarcoma (*n* = 104; 29%), liposarcoma (*n* = 40; 11%), and synovial sarcoma (*n* = 12; 3%); the remainder (*n* = 207; 57%) comprised 27 histologic subtypes. Treated patients were younger and had lower ECOG scores; 75% and 25% received first-line combination or monotherapy, respectively. Median OS of treated and untreated patients was 22 and 17 months, respectively, and 29 months in patients with the three most common subtypes. Before controlling for effects of covariates, younger age and lower ECOG scores were associated with better OS and PFS. *Conclusion*. This study provides insights into mSTS epidemiology, treatment patterns, and outcomes in a large community-based oncology network. These results warrant further studies with larger cohorts.

## 1. Introduction 

Soft tissue sarcomas (STS) are rare mesenchymal tumors that account for 1% of adult cancers [1–7] and comprise over 50 different histologic subtypes that differ in pathogenesis and outcomes [1–3, 6, 7]. Collectively, they are associated with a mortality rate of over 4,000 patients per year [2]. The treatment of STS is dependent upon several factors, including histologic subtype, disease stage, and patient performance status; the treatment options include surgery, radiotherapy, and/or chemotherapy [1, 3, 6–10]. Although localized resected disease can often be cured, the prognosis of patients with metastatic STS (mSTS) remains poor, with median survival of approximately one year [1, 3, 6, 11–15]. Good prognostic factors for mSTS include younger patients with good performance status and low tumor grade [3, 6, 16].

Most of our insights regarding factors that influence the outcomes following chemotherapy in mSTS have been obtained mainly from clinical trials [11–14, 16]. The purpose of this study was to gain an improved understanding of the “realworld” epidemiology as well as treatment patterns and outcomes of mSTS in the setting of community oncology clinics.

## 2. Methods

Data was obtained from the McKesson Specialty Health (MSH)/US Oncology (USON) iKnowMed (iKM) electronic health record (EHR) and electronic chart review. The study period was from July 1, 2007, to June 30, 2010, with follow-up through June 30, 2011. Adults (≥18 years) with mSTS were included if they received care at practice sites with the full capabilities of the iKM EHR system and had at least two visits during the study period. Patients were excluded if during the study period they were diagnosed with or treated for a primary cancer other than mSTS or were enrolled in a randomized clinical trial. Electronic chart review was conducted to validate and supplement EHR data for critical parameters, including cell morphology and histology. For the measurement of progression-free survival (PFS), an escalation in the line of therapy (LOT) from first-line to second-line was used as a proxy measurement for disease progression. Thus, PFS in this study was defined as the time in months from the initiation of first-line chemotherapy to the initiation of second-line chemotherapy, or death from any cause, whichever occurred first. Patients who were progression-free were censored at the end of the study follow-up period or at the date they were last known to be alive, whichever occurred first.

Descriptive analyses were conducted on patient demographics and clinical characteristics, as well as treatment patterns. Overall survival (OS) and PFS were estimated using Kaplan-Meier plots. Mean and median survival time and survival rates were derived with 95% confidence intervals (CI). SAS 9.2 and STATA 11.2 were used for data management and statistical analysis.

## 3. Results

### 3.1. Study Population

In the iKnowMed EHR, 4,245 individuals had histologically confirmed STS, of which 1,286 (30%) had metastatic disease. Supplementary Figure 1 depicts the patient consort diagram; based upon the inclusion/exclusion criteria employed, we selected 363 mSTS patients for further evaluation: 174 of the 329 treated and 189 of the 957 untreated patients (see Supplementary Material available online at http://dx.doi.org/10.1155/2014/145764).

Table 1 describes the demographic and clinical characteristics of our study sample. The mean age (standard deviation) at diagnosis of mSTS in the entire study population was 61 (16) years. Forty-eight percent were female and 52% were male. Based on the histology of their tumors, the study population (*n* = 363) was categorized into four major groups: those with leiomyosarcoma (*n* = 104; 29%), liposarcoma (*n* = 40; 11%), and synovial sarcoma (*n* = 12; 3%), and the remainder were designated as “other” (*n* = 207; 57%) as they comprised subjects with 27 histologic subtypes, each with small sample sizes. The frequency of the histology subtypes was similar in treated and untreated patients (Table 1). We adopted this categorization schema as these were the most prevalent groups based on histology, and recent studies used similar groupings [17]. The most common primary tumor sites in the treated population were the extremities (33%), retroperitoneal (19%), and the trunk and viscera (14%).

Among treated and untreated patients with available clinical data, presentation in both groups was most commonly with stage IV disease (54% and 67%, resp.), grade 3 tumor (69% each), resectable tumor (50% and 61%, resp.), and ECOG score of 1 (49% and 47%, resp.) (Table 1). Treated patients were younger (median = 58 yr) than untreated patients (median = 65 yr) and the overall ECOG performance at study entry between treated and untreated patients differed (Table 1). In addition, male patients were more likely to receive chemotherapy when compared with female patients (60% versus 40%, resp.).

### 3.2. Treatment Patterns

Of the 174 treated patients, 173 (99%) and 42 (24%) received first- and second-line therapies, respectively, and only 14 (8%) continued with third-line therapy. (Note: one of the 174 treated patients received second-line therapy as initial therapy.) The most frequently used chemotherapy regimens in first-, second-, and third-line therapies were as follows: for first-line chemotherapy, doxorubicin plus ifosfamide (29%), docetaxel plus gemcitabine (24%), or doxorubicin alone (12%) was used. For second-line chemotherapy, docetaxel plus gemcitabine (52%) was used the most, followed by doxorubicin alone (17%). For third-line chemotherapy, liposomal doxorubicin alone (29%) or docetaxel plus gemcitabine (21%) was given.

Of the treated patients, 64 (37%) received monotherapy, whereas 135 (78%) received combination therapy; approximately 14% received both monotherapy and combination therapies. Among those receiving first-line therapy (*n* = 173), 28% received monotherapy and 72% combination therapy, and similar proportions were observed for those receiving second-line therapy (29% and 71%, resp.). Of the 14 patients who continued chemotherapy into third-line, 57% and 43% received monotherapy and combination therapy, respectively. These data suggested that among those receiving first- or second-line chemotherapies, a greater proportion received combination compared with monotherapy. In contrast, the reverse pattern was observed in those receiving third-line therapy.

The following treatment patterns were observed. Among the 49 patients who received monotherapy as first-line therapy, doxorubicin (43%), gemcitabine (27%), and liposomal doxorubicin (14%) were the more frequently used agents, while among the 126 patients receiving first-line combination therapy the most common regimens were doxorubicin plus ifosfamide (41%), docetaxel plus gemcitabine (35%), and doxorubicin plus dacarbazine (6%). Among the 12 patients who received monotherapy as second-line therapy, doxorubicin (58%) was most often used, while in the 30 subjects receiving combination second-line therapy, docetaxel plus gemcitabine (73%) was used most frequently. Ninety-five patients received anthracycline-containing first-line therapy, and of these 37 continued with second-line chemotherapy; among these patients docetaxel plus gemcitabine (70%) was most commonly used.

We examined the usage of monotherapy versus combination therapy according to histology subtype (Supplementary Table 1). Among those receiving combination (*n* = 129) and monotherapy (*n* = 50), 56% and 40%, respectively, were patients with the top three subtypes. Reflecting that a lower proportion of patients with the other subtypes received combination therapy, 72 of the 92 subjects with the top three subtypes (78.3%) versus 57 of the 87 individuals with the other STSs (65.5%) received combination chemotherapy (Supplementary Table 1).

Among the 50 individuals receiving first-line monotherapy, 28% completed therapy as scheduled, and early discontinuation of therapy was attributable to disease progression in 18% and drug toxicity in 16%. Among the 129 subjects receiving first-line combination therapy, the corresponding values were 35%, 16%, and 13%, respectively. Twelve subjects received second-line monotherapy, and of these 33% completed therapy, 25% discontinued therapy early because of disease progression, and 17% terminated therapy early because of drug toxicity; the corresponding proportions for the 30 patients who received second-line combination chemotherapy were 27%, 17%, and 7%, respectively.

### 3.3. OS and PFS: Unstratified Analyses

Approximately 40% of the treated and untreated patients died during follow-up. The median OS of treated patients was 22 (95% CI, 17 to 29) months whereas for the untreated patients it was 17 (95% CI, 11 to 23) months (Supplementary Table 2). The percent OS of treated patients at 6, 12, 24, and 36 months was 88%, 69%, 45%, and 32%, respectively, while that of untreated patients was 70%, 55%, 41%, and 34%, respectively (Supplementary Table 2).

**Figure 1:**
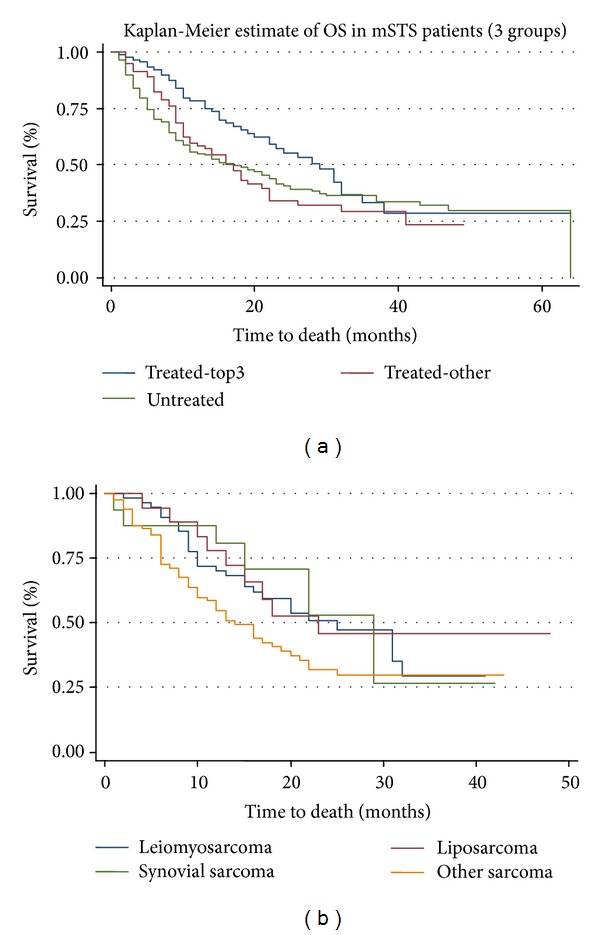
Kaplan-Meier estimates of overall survival in untreated and treated metastatic STS patients. (a) Treated patients were stratified according to histologic subtype. The top three histology subtypes were pooled into one group (treated-top 3), and the remainder were classified as “treated-other.” (b) Kaplan-Meier plots for the three most common STS subtypes (leiomyosarcoma, liposarcoma, and synovial sarcoma) in the study population and the remaining subtypes (other sarcoma).

The OS estimates of patients stratified by STS subtype are shown in Supplementary Table 3 and depicted in Figure 1.  Figure 1(a) shows the Kaplan-Meier plots for the OS of two treated subgroups and untreated patients, and Figure 1(b) shows the Kaplan-Meier plots of OS for each of the top three histology subtypes and the other sarcomas. The Kaplan-Meier plots for the top three subtypes were not different from each other (Figure 1(b)) but were different from the other sarcomas and the untreated patients (Figures 1(a) and 1(b)). The median OS for the treated patients with the top three most common histologic subtypes was 29 (95% CI, 22 to 35) months. The median for the treated patients with the other histologic subtypes was 17 (95% CI, 11 to 22) months, similar to that observed in the untreated patients.

The PFS estimates of patients stratified by STS subtype are shown in Supplementary Table 4 and depicted in Supplementary Figure 2. The median overall PFS of treated patients was 11 (95% CI, 9 to 14) months. The median PFS for treated patients with leiomyosarcoma, liposarcoma, synovial sarcoma, or other sarcomas was 12, 18, 17, and 7 months, respectively (Supplementary Table 4).

### 3.4. OS and PFS: Stratified by Line of Therapy

The median OS of patients treated with first-line chemotherapy during the study period was 22 (95% CI, 17 to 28) months. The mean OS was 24 (95% CI, 24 to 24) months, and the 6-, 12-, 24-, and 36-month OS rates for first-line therapy were 88% (95% CI, 82% to 92%), 69% (95% CI, 62% to 76%), 45% (95% CI, 36% to 53%), and 33% (95% CI, 24% to 42%), respectively. The median OS of patients treated with second-line therapy was 11 (95% CI, 8 to 19) months, whereas the mean OS was 13 (95% CI, 12 to 13) months and the 6- and 12-month OS rates were 70% (95% CI, 54 to 82) and 44% (95% CI, 29 to 58%), respectively.

The median PFS of patients treated with first-line chemotherapy during the study period was 11 (95% CI, 9 to 14) months. The mean PFS was 15 (95% CI, 14 to 15) months, and the 6-, 12-, 24- and 36-month PFS rates for first-line therapy were 66% (95% CI, 58% to 72%), 46% (95% CI, 38% to 54%), 28% (95% CI, 20% to 36%), and 17% (95% CI, 10% to 26%), respectively (Supplementary Table 4). The median PFS of patients treated with second-line therapy was 9 (95% CI, 7 to 12) months, whereas the mean PFS was 11 (95% CI, 10 to 11) months, and the 6- and 12-month PFS rates were 64% (95% CI, 47% to 77%) and 35% (95% CI, 20% to 50%), respectively.

### 3.5. Overall Survival by Age

Among those receiving first-line therapy, 102 (58.9%), 39 (22.5%), and 25 (14.5%) were <65, 65–75, and ≥75 years old, respectively (age was unknown in 7 subjects (4.1%)), and the corresponding values for those receiving second-line therapy were 26 (61.9%), 9 (21.4%), and 3 (7.1%) (age was unknown in 4 (9.5%) subjects). Among those receiving first-line monotherapy (*n* = 50), 40%, 20%, and 36% were <65, 65–75, and ≥75 years old, respectively, and corresponding values for those receiving first-line combination therapy (*n* = 129) were 65.9%, 22.5%, and 7.8%, respectively. There was a similar age distribution by second-line monotherapy and combination chemotherapy.

**Figure 2:**
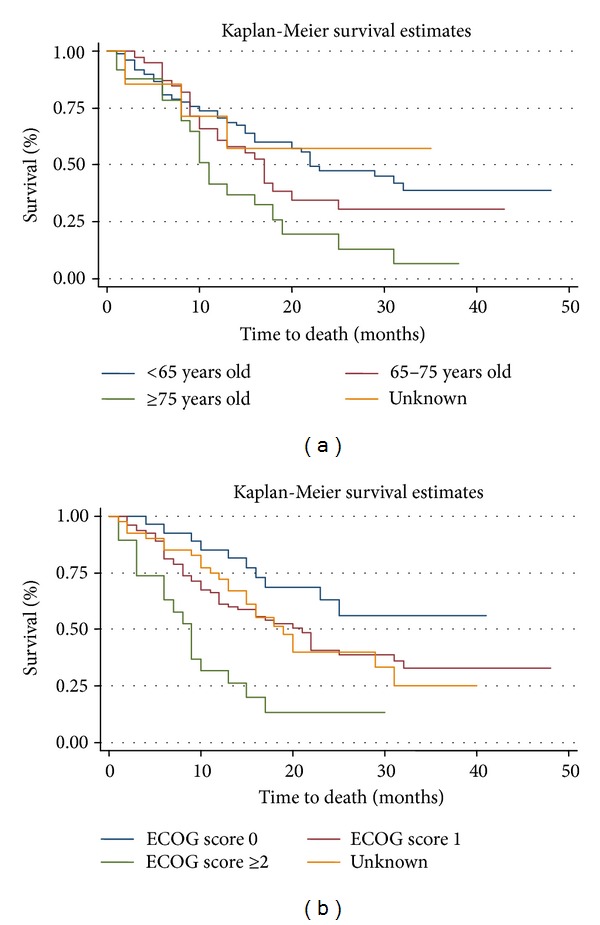
Overall survival by age and ECOG status among mSTS patients starting first-line therapy. (a) Kaplan-Meier plots by age group. (b) Kaplan-Meier plots by baseline ECOG status.

The Kaplan-Meier analyses for OS by age at the start of first-line therapy are shown in Figure 2(a). The mean OS for those initiating first-line therapy at ages <65, 65 to 75, and >75 years was 21 (95% CI, 21 to 21), 16 (95% CI, 16 to 16), and 13 (95% CI, 13 to 14) months, respectively. The mean PFS for those initiating first-line therapy at ages <65, 65–75, and >75 years was 15 (95% CI, 15 to 15), 14 (95% CI, 14 to 15), and 12 (95% CI, 11 to 13) months, respectively (data not shown).

### 3.6. Overall Survival by ECOG Status

Among those receiving first-line therapy, 29 (16.7%), 83 (47.7%), and 19 (10.9%) had an ECOG status of 0, 1, and 2, respectively (ECOG status was unknown in 42 subjects (24.1%)). The Kaplan-Meier analyses of OS by baseline ECOG status are shown in Figure 2(b). The mean OS for those initiating first-line therapy by ECOG status of 0, 1, and ≥2 was 21 (95% CI, 20 to 21), 20 (95% CI, 20 to 20), and 9 (95% CI, 9 to 10) months, respectively. The corresponding mean PFS values were 17 (95% CI, 16 to 18), 15 (95% CI, 15 to 15), and 8 (95% CI, 7 to 8) months, respectively.

Among those receiving first-line monotherapy (*n* = 50), 6%, 50%, and 20% had ECOG scores of 0, 1, and ≥2, respectively, and the corresponding values for those receiving first-line combination therapy were 20.9%, 46.5%, and 6.9%, respectively. The relative distribution of ECOG scores by second-line monotherapy and combination chemotherapy was similar.

### 3.7. Overall Survival by Metastasis Site

Among subjects who initiated first-line therapy, the most common sites for metastasis were lung (36.9%), trunk and viscera (10.4%), retroperitoneal (8.6%), and multiple metastatic sites (8.6%). The mean OS in these patients was 15 (95% CI, 14 to 15), 16 (95% CI, 15 to 16), 17 (95% CI, 16 to 17), and 20 (95% CI, 20 to 21) months, respectively. The mean PFS was 10 (95% CI, 10 to 10), 14 (95% CI, 14 to 15), 14 (95% CI, 14 to 15), and 17 (95% CI, 17 to 18) months, respectively (data not shown).

## 4. Discussion

This retrospective observational study conducted in a large community oncology network yielded six key findings. First, STS histologic subtypes were similar between the treated and untreated subjects (i.e., tumor subtypes, stage, and grade). Leiomyosarcoma, liposarcoma, and synovial sarcoma were the top three most common histologic subtypes in this patient population, consistent with the previously reported distribution patterns [7, 15, 16]. Second, while tumor characteristics were similar, treated patients differed from untreated individuals in three respects: they were younger, a greater proportion was men, and they had lower ECOG scores.

Third, our study revealed insights into chemotherapy treatment patterns for mSTS in the community setting. Among patients receiving first-line chemotherapy, ~75% and ~25% received combination and monotherapy, respectively. Among patients with leiomyosarcoma, the most frequent form of mSTS in our study population, 84% received combination therapy. The most common combination regimens in the overall study population were doxorubicin plus ifosfamide (29%) and docetaxel plus gemcitabine (24%). This choice is expected based on prevailing clinical practice [18]. For example, gemcitabine with docetaxel has been found to be active in leiomyosarcoma of uterine and gastrointestinal origin: a phase 2 study reported a higher response rate in this subtype for combination docetaxel plus gemcitabine versus gemcitabine alone (32% versus 27%, resp.) and significantly improved progression-free survival (6.3 versus 3 months, resp.) [19]. Although the benefits of chemotherapy for mSTS and the use of combination versus single agent chemotherapy for mSTS are unclear, combination chemotherapy is generally an accepted practice standard in the USA [2, 3]. Further research is needed to determine the optimal dosing and tolerability of these regimens in these patients. Even though the primary focus of this study was the chemotherapy treatment patterns in the community setting, future research should also explore the effects of surgery and radiation on these patients.

Fourth, the median OS of treated and untreated patients prior to accounting for tumor subtype was 22 and 17 months, respectively. A retrospective analysis of seven clinical trials of chemotherapy-naïve patients with advanced STS revealed that the overall median survival time of the 2,185 patients in the therapy arms was 51 weeks [16]; similar data were observed in more recent clinical trials [15, 17]. The basis for the longer overall survival times in our study subjects treated in the community compared with results from clinical trials is unclear and needs further investigation. However, consistent with previous studies [15, 16], patients with the top three histologic subtypes had better outcomes than those with the other subtypes. Among patients with the top three histologic subtypes, the median overall survival was 29 months, with similar trends observed for PFS. This observation could relate to differences in the underlying biology of these three tumors, such that, compared to the heterogeneous group of STS that were pooled into one group (other), the top three histologic types may be more responsive to therapy and/or have less aggressive disease characteristics. Another possibility could be that a slightly greater proportion of patients with the top three subtypes were treated with combination therapy (78.3%) compared to those with the other STS subtypes (65.5%). Selection biases could have contributed to these differences in outcomes by histology subtype. However, this was less likely, as we found that ages and ECOG scores between treated patients were similar by histology subtype.

Fifth, consistent with prior studies [16], we also found that younger age and lower ECOG scores were associated with longer OS in treated patients. While there was a significant difference in mean age and baseline ECOG scores between treated and untreated patients, larger cohorts in future studies will be needed to properly control for the influence these factors may have on OS and PFS outcomes. Finally, lung was the most common site for metastasis, and those with lung metastasis had shorter OS and PFS compared to subjects with metastasis to other sites.

Due to the retrospective, observational design of this study, there are some limitations worth noting. While the use of a large geographically dispersed cohort of community-based patients provides confidence that our results may potentially be able to be generalized, patterns of care within the MSH/USON network may differ to some extent from community-based treatment patterns in general. This may be due to the encouragement given to oncologists by the MSH/USON network administration to base their therapy decisions on evidence-based treatment guidelines. Additional limitations of this study include the exclusion of patients from specific sites in the MSH/USON network from the study sample because only partial iKnowMed EHR capabilities were adopted at these sites; it is possible that patterns of care at these specific sites may differ to some extent from the remainder of the sites. Another limitation includes the lack of differentiation of STS subtypes. Our EHR and chart review did not capture the different variants of liposarcoma or other important subtypes. Selection bias of subtype variants may have influenced response and survival rates and should be considered in future research. Also, escalation in LOT was used as a proxy for disease progression. Since there may be some delay from disease progression to when patients received their next line of chemotherapy, the progression-free survival may be overestimated. In addition, the iKnowMed data are collected for clinical practice reasons and not for research purposes. This may limit the standardization of the data collection methods and instruments as well as the reporting practices of the physician. Finally, our study was not designed to compare the efficacy of monotherapy versus combination therapy or determine the factors that associate with poorer clinical outcomes in STS. Thus, the inferences of this observational study need confirmation in randomized, controlled trials of adequate size prospectively designed to address these questions.

## 5. Conclusions

To our knowledge, this is the first study to use a cancer-specific database to capture “realworld” clinical data on patients with mSTS in the community-based setting. The results of this study are strengthened by the large sample size and potentially greater diversity of care compared with clinical trial or tertiary care academic settings. Taken together, by examining a community-based, cancer-specific EHR, we provide new insights into the epidemiology, treatment patterns, and outcomes of patients with mSTS who received care outside of an academic or clinical trial setting in the USA and elaborate on their implications for future clinical research.

## Conflict of Interests

Employment or Leadership Position: E. Nadler (The US Oncology Network). Employment or Leadership Position: R. Borker and M. Hackshaw (GlaxoSmithKline). Consultant or Advisory Role: E. Nadler (The US Oncology Network). Stock Ownership: R. Borker and M. Hackshaw (GlaxoSmithKline). Honoraria: None. James Ewing, MD, and Eric Nadler, MD, are Medical Directors of Health Informatics at US Oncology Research, McKesson Specialty Health, Department of Oncology, Baylor Charles A. Sammons Cancer Center and Baylor University Medical Center at Dallas. Rohit Borker and Michelle D. Hackshaw are employees of GlaxoSmithKline.

## Supplementary Material

Supplementary material included online only includes the following figures and tables: Figure 1 depicts the patient consort diagram. Table 1 shows the use of monotherapy versus combination therapy according to histology subtype. Among those receiving combination and monotherapy, 56% and 40%, respectively, had the top 3 subtypes. Table 2 depicts overall survival and progression-free survival. Approximately 40% of treated and untreated patients died during follow-up. Median overall survival of treated patients was 22 months and for untreated patients it was 17 months. Table 3 shows overall survival estimates of patients stratified by STS subtype, and Table 4 and Figure 2 show progression-free survival stratified by the STS subtypes leiomyosarcoma, liposarcoma, synovial sarcoma, and other.

## Figures and Tables

**Table 1:** Demographic and clinical characteristics of treated and untreated metastatic STS patients.

Characteristic	Total (*N* = 363)	Treated patients (*N* = 174)	Untreated patients (*N* = 189)	*P*
Age at treatment, *N* (%)				
Mean (SD)	61 (16)	58 (14)	63 (17)	0.0031
Median (range)	62 (18, 91)	58 (19, 90)	65 (18, 91)	0.0005
<65	203 (60)	109 (63)	94 (50)	<0.0001
65–75	76 (21)	42 (24)	34 (18)	
≥75	84 (23)	23 (13)	61 (32)	
Gender, *N* (%)				
Female	175 (48)	70 (40)	105 (56)	0.0035
Male	188 (52)	104 (60)	84 (44)	
BMI, *N* (%)				
Mean (SD)	28.1 (6.3)	28.5 (5.9)	27.8 (6.8)	0.2742
Median (range)	26.8 (15.4, 52.1)	27.5 (17.1, 49.5)	26.4 (15.4, 52.1)	0.0624
Underweight	6 (2)	3 (2)	3 (2)	0.1270
Normal weight	104 (31)	43 (25)	61 (37)	
Overweight	126 (37)	70 (40)	56 (34)	
Obese	103 (30)	57 (33)	46 (28)	
Missing	24	1	23	
Stage at diagnosis*, *N* (%)				
I	19 (8)	12 (10)	7 (6)	0.1253
II	19 (8)	9 (7)	10 (9)	
III	56 (24)	35 (29)	21 (18)	
IV	142 (60)	65 (54)	77 (67)	
Missing	127	53	74	
Cell morphology, *N* (%)			
Leiomyosarcoma	104 (29)	50 (29)	54 (29)
Liposarcoma	40 (11)	20 (11)	20 (11)
Synovial Sarcoma	12 (3)	9 (5)	3 (2)
Other STS**	207 (57)	95 (55)	112 (59)
Tumor grade, *N* (%)				
1	24 (12)	11 (10)	13 (13)	0.8523
2	33 (16)	19 (18)	14 (14)	
3	142 (69)	74 (69)	68 (69)	
4+	8 (4)	4 (4)	4 (4)	
Missing	156	66	90	
Tumor type, *N* (%)				
Resectable	105 (61)	51 (50)	54 (61)	0.8543
Unresectable	68 (39)	34 (40)	34 (39)	
Missing	190	89	101	
Baseline ECOG, *N* (%)				
0	59 (20)	30 (22)	29 (18)	0.0034
1	153 (52)	79 (49)	74 (47)	
2+	84 (28)	25 (19)	59 (36)	
Missing	67	40	27	
ECOG after first-line treatment, *N* (%)				
0	17 (15)	17 (15)	
1	69 (59)	69 (59)	
2+	30 (26)	30 (26)	
Missing	58	58	
Primary site^†^				
Head and neck		14 (8)	
Lung		9 (5)	
Liver		3 (2)	
Trunk and viscera		25 (14)	
Retroperitoneal		33 (19)	
Extremity		58 (33)	
Other		29 (17)	
Missing		3 (2)	

SD: standard deviation, BMI: body mass index, and ECOG: Eastern Cooperative Oncology Group Performance Status. *P*: significance value by Chi-square test.

*The stage of disease for each patient is consistent with the descriptions of the AJCC7 classifications of disease.

**Other STS include angiosarcoma of soft tissue, alveolar rhabdomyosarcoma, alveolar soft part sarcoma, fibrosarcoma, Kaposi sarcoma, PNET, pleomorphic rhabdomyosarcoma, clear-cell sarcoma of soft tissue, malignant fibrous histiocytoma, myxofibrosarcoma, malignant phyllodes cystosarcoma, embryonal rhabdomyosarcoma, extraskeletal Ewing tumor, extraskeletal myxoid chondrosarcoma, osteosarcoma, malignant ossifying fibromyxoid tumor, malignant peripheral nerve sheet tumor, hemangiopericytoma, and sarcoma NOS.

^†^Primary sites were captured through chart reviews for the treated patient cohort (*N* = 174) only. Chart reviews were not conducted for the untreated patient cohort (*N* = 189).
