# Supplementary material for: Epidemiology, Treatment Patterns, and Outcomes of Metastatic Soft Tissue Sarcoma in a Community-Based Oncology Network
Source: Sarcoma. 2014 Jan 12;2014:145764. doi: 10.1155/2014/145764 (PMC3942092; doi:10.1155/2014/145764)
Supplement: Supplementary file 1 — Supplementary material included online only includes the following figures and tables: Figure 1 depicts the patient consort diagram. Table 1 shows the use of monotherapy versus combination therapy according to histology subtype. Among those receiving combination and monotherapy, 56% and 40%, respectively, had the top 3 subtypes. Table 2 depicts overall survival and progression-free survival. Approximately 40% of treated and untreated patients died during follow-up. Median overall survival of treated patients was 22 months and for untreated patients it was 17 months. Table 3 shows overall survival estimates of patients stratified by STS subtype, and Table 4 and Figure 2 show progression-free survival stratified by the STS subtypes leiomyosarcoma, liposarcoma, synovial sarcoma, and other. [file 145764.f1.pdf]

Online only:

Supplementary Figure 1. Patient consort diagram outlining inclusion and exclusion criteria used for selection of patients for study.

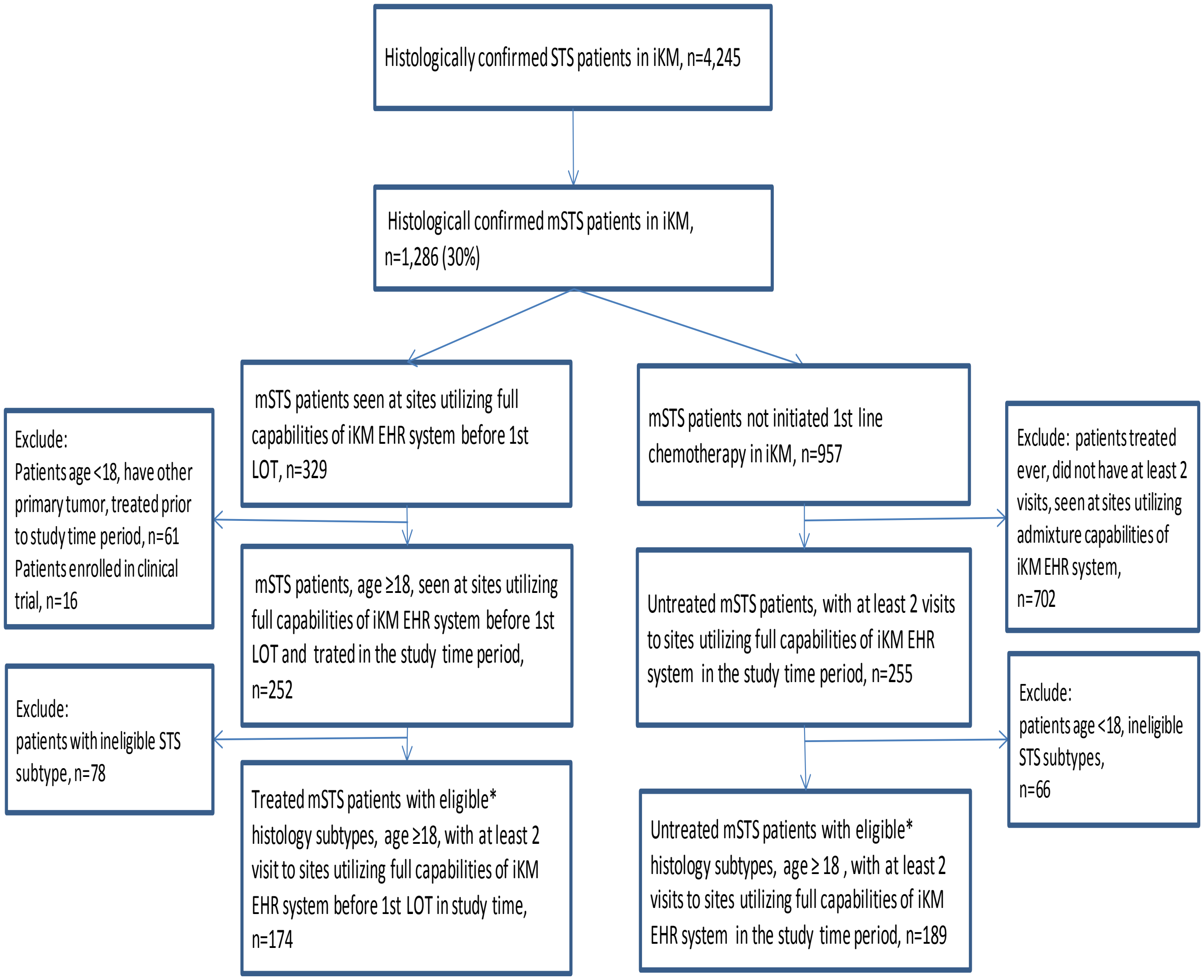

*Eligible histology subtypes include leiomyosarcoma, liposarcoma, synovial sarcoma, and other STS as described in the footnote to Table 1.

**LOT, line of therapy

Supplementary Figure 2. Kaplan-Meier estimates for PFS in treated patients according to the three most common STS subtypes in the study population and the remaining STSs (other sarcoma).

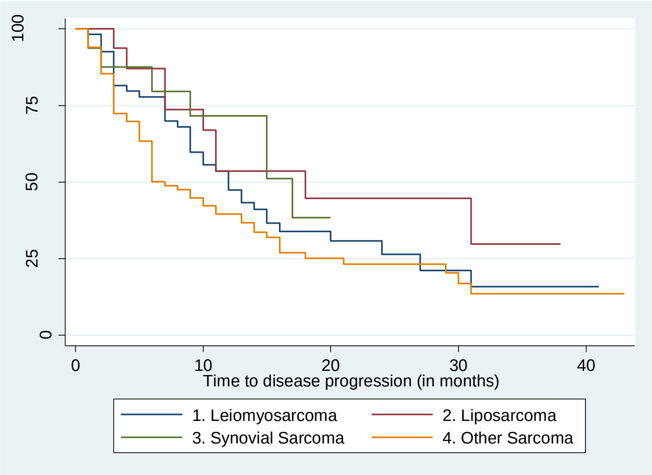

*Among 173 patients receiving first-line chemotherapy, 50 received monotherapy, and 129 received combination therapy.

**Other types of STS are described in the footnote to Table 1.

Supplementary Table 1. Monotherapy and combination therapy by subtype/cell morphology of metastatic STS.

	Monotherapy (N=50)		Combination Therapy (N=129)*	
Subtype/Cell Morphology of STS	N (%)	95% CI	N (%)	95% CI
Leiomyosarcoma	9 (18.0)	(7.4, 28.7)	47 (36.4)	(28.1, 44.7)
Liposarcoma	8 (16.0)	(5.8, 26.2)	12 (9.3)	(4.3, 14.3)
Synovial sarcoma	3 (6.0)	(0.0, 12.6)	13 (10.1)	(4.9, 15.3)
Other sarcoma**	30 (60.0)	(46.4, 73.6)	57 (44.2)	(35.6, 52.8)

*Among 173 patients receiving first-line chemotherapy, 50 received monotherapy, and 129 received combination therapy.

**Other sarcomas are outlined in the footnote for Table 1.

Supplementary Table 2. Overall survival in months for treated and untreated metastatic STS patients.

	Median (95% CI)	Mean (95% CI)	6-Month Survival (%), (95% CI)	12-Month Survival (%), (95% CI)	24-Month Survival (%), (95% CI)	36-Month Survival (%), (95% CI)
Overall* (N=354)	20 (16, 31)	29 (29, 29)	79 (74, 83)	62 (57, 67)	43 (37, 49)	35 (29, 41)
Treated (N=174)	22 (17, 29)	23 (22, 23)	88 (82, 92)	69 (61,75)	45 (38, 54)	32 (23, 41)
Untreated* (N=180)	17 (11, 23)	28 (27, 28)	70 (62, 77)	55 (47, 62)	41 (33, 49)	34 (26, 42)

*9 untreated patients were removed from the analysis because of missing data.

Supplementary Table 3. Overall survival in months of treated and untreated metastatic STS patients stratified by subtype.

	Median Months, (95% CI)	Mean Months, (95% CI)	6-Month Survival (%), (95% CI)	12-Month Survival (%), (95% CI)	24-Month Survival (%), (95% CI)	36-Month Survival (%), (95% CI)
Overall (N=354)	20 (16, 31)	29 (29, 29)	79 (74, 83)	62 (57, 67)	43 (37, 49)	35 (29, 41)
*Treated-top 3 histology subtypes (N=91)	29 (22, 35)	25 (24, 25)	92 (84, 96)	75 (64, 83)	55 (43, 66)	33 (20, 47)
**Treated-other sarcomas (N=83)	17 (11, 22)	20 (20, 21)	83 (72, 89)	58 (47, 68)	32 (21, 43)	29 (19, 41)
Untreated† (N=180)	17 (11, 23)	28 (28, 28)	70 (62, 77)	55 (47, 62)	41 (33, 49)	34 (26, 42)

*Treated-top 3 histology subtypes and **other sarcomas are described in the footnote to Table 1.

†9 untreated patients were removed from the analysis because of missing data.

Supplementary Table 4. Progression-free survival in months for treated metastatic STS patients stratified by subtype.

	Median Months, (95% CI)	Mean Months, (95% CI)	6-Month Survival (%), (95% CI)	12-Month Survival (%), (95% CI)
Overall (N=173*)	11 (9, 14)	15 (14, 15)	66 (58, 72)	46 (38, 54)
Leiomyosarcoma (N=55)	12 (9, 15)	15 (15, 15)	70 (56, 80)	47 (33, 60)
Liposarcoma (N=19)	18 (7, -†)	19 (17, 21)	74 (44, 89)	54 (27, 75)
Synovial sarcoma (N=16)	17 (6, -)	13 (12, 14)	80 (49, 93)	72 (40, 88)
Other sarcomas** (N=83)	7 (6, 13)	13 (12, 13)	50 (39, 61)	40 (29, 50)

*Among those 174 patients, one patient did not receive first-line treatment; they received second-line treatment as initial therapy.

**Other sarcomas are described in the footnote to Table 1.

PFS in this study was defined as the time in months from the initiation of first-line chemotherapy to the initiation of second-line chemotherapy, or death from any cause, whichever occurred first. The mean PFS and its standard error were underestimated because the largest observation was censored and the estimation was restricted to the longest event time.
